# Notes and News

**Published:** 1904-02-06

**Authors:** 


### NOTES AND NEWS.

THE King has become an annual subscriber of £10 10s. to the Metropolitan Convalescent Institution.

THE late Mr. George Herbert of Carshalton has bequeathed £1,000 to the Carshalton Cottage Hospital.

IN the Florida Navy Yard at Pensacola an experimental tuberculosis camp has been established. It is proposed to start similar naval sanatoria elsewhere.

A COMPLIMENTARY dinner will be given to Mr. Timothy Holmes on his resignation of the office of treasurer to St. George's Hospital which he has occupied for so long. The dinner will be held at the Hôtel Métropole on February 13th. The dinner may be attended by any friend of Mr. Timothy Holmes, and all particulars can be obtained from the Secretary of the Hospital.

THE South African Medical Congress has once more resumed its meeting after the long interval caused by the war. The recent Congress was held at Capetown. Some excellent papers were read and considerable interest taken in the discussions. One outcome of the meeting is that it has been decided definitely to form a Medical Guild for South Africa, and Dr. Fuller further strongly urged the establishment of a South African Medical School. The latter suggestion was met by very divided opinions.

WE announced in a recent issue the intention of Battersea to institute instruction in hygiene and the care of infants for the benefit of poor mothers of the district. Manchester possesses a society which works on the proposed lines and is meeting with success. By virtue of the ministrations of the society it is claimed that infant mortality has already decreased. The visitors, of whom there are 23 at present, visit the homes of the poor and give practical instruction and advice. The difficulty must be to find persons of sufficient discrimination, intelligence, and tact to undertake the work, which, if effectively carried out must be of great benefit to the whole community.

THE Anglo-American Hospital at Cairo was opened by Lord and Lady Cromer on January 21st.

THE second number of the "British Journal of Children's Diseases" maintains the high standard upon which we commented in respect of the first issue. Dr. Henry Ashley is the writer of the first of many interesting and valuable contributions.

THE Tuberculosis Commission of Maryland, United States, will open an exhibition to display the results of the investigation of the Commission. Reports will be given as to the means taken throughout the State to enlighten the community as to the means of preventing or limiting the spread of the disease, and not the least interesting exhibit will be one of photographs of the special haunts of consumption in large cities.

THE importance to the community of calling attention to the physical condition of the young is becoming universally recognised. England is by no means a pioneer in these questions, and school hygiene especially claims much more careful attention in her hands than it has yet obtained. In Philadelphia a system of regular school inspection has been instituted, and the Board of Education is arranging a series of lectures on school hygiene for the teachers.

THE use of the Roentgen rays appears to be a new danger for medical men. In Chicago a patient has sued his medical attendant for 25,000 dollars damages for burning his face with the rays and preventing the growth of beard or moustache on one side of his face. Had the mishap occurred in connection with a member of the Bar in England the doctor might have been regarded as a benefactor by a busy barrister relieved of the necessity to shave both sides of his face.

MEDICAL matters in Chicago show considerable activity at the present time. Three new clinical departments—for medicine, surgery and obstetrics—are to be added to the medical department of the University; also biological laboratories and a temporary dispensary. Five new hospitals for medicine, surgery, obstetrics, the diseases of children, and contagious diseases are also contemplated, as well as the establishment of a school for dentistry. Apart from the University, the Michael Reese Hospital is to be rebuilt.

THE section appertaining to hospitals at the forthcoming World's Fair Exhibition in St. Louis, United States, will be situated in the department devoted to social economy. The exhibits relating to hospitals, asylums, and child-saving institutions will be classified together. The pathological section will include statistics, exhibits, slides, photographs, and illustrations demonstrating statistical and other facts. In the section devoted to the prevention of disease there will be illustrated special laboratories of research and records of administrative methods in relation to boards of health, sanitary inspection, disinfection, and education. The hospital section will include illustrations of hospitals. At the Congress of Arts and Sciences in connection with the Exhibition discussions on all subjects connected with the exhibits will take place.

